# Mean corpuscular volume as a prognostic factor for 30-day mortality in major trauma patients: a retrospective cohort study

**DOI:** 10.1038/s41598-024-54057-1

**Published:** 2024-02-17

**Authors:** Hanlim Choi, Jin Young Lee, Younghoon Sul, Se Heon Kim, Jin Bong Ye, Jin Suk Lee, Soo Young Yoon, Junepill Seok, Jung Hee Choi

**Affiliations:** 1 Department of Surgery, College of Medicine, Chungbuk National University, Cheongju, Republic of Korea; 2 Deparment of Trauma Surgery, Trauma Center, Chungbuk National University Hospital, 776, 1 Sunhwan-ro, Seowon-gu, Cheongju, 28644 Republic of Korea; 3 Department of Trauma Surgery, College of Medicine, Chungbuk National University, Cheongju, Republic of Korea; 4 Department of Cardiovascular and Thoracic Surgery, Trauma Center, Chungbuk National University Hospital, Cheongju, Republic of Korea; 5 Department of Anesthesiology and Pain Medicine, Chungbuk National University Hospital, Cheongju, Republic of Korea

**Keywords:** Risk factors, Predictive markers

## Abstract

We investigated the clinical implications of the mean corpuscular volume (MCV) in patients with major trauma. This single-center retrospective review included 2021 trauma patients admitted to the intensive care unit between January 2016 and June 2020. We included 1218 patients aged $\ge$ 18 years with an injury severity score $\ge$ 16 in the final analysis. The clinical and laboratory variables were compared between macrocytosis (defined as MCV $\ge$ 100 fL) and non-macrocytosis groups. Cox regression analysis was performed to calculate the hazard ratios (HRs) of variables for 30-day mortality, with adjustment for other potential confounding factors. The initial mean value of MCV was 102.7 fL in the macrocytosis group (n = 199) and 93.7 fL in the non-macrocytosis group (n = 1019). The macrocytosis group showed a significantly higher proportion of initial hypotension, transfusion within 4 and 24 h, and 30-day mortality than the non-macrocytosis group. Age ($\ge$ 65 years), hypotension (systolic blood pressure $\le$ 90 mmHg), transfusion (within 4 h), anemia (Hb < 12 g/day in women, < 13 g/day in men), and macrocytosis were significantly associated with 30-day mortality (adjusted HR = 1.4; 95% confidence interval 1.01–1.94; *p* = 0.046) in major trauma patients. Thus, **i**nitial macrocytosis independently predicted 30-day mortality in patients with major trauma at a Level I trauma center.

## Introduction

Anemia is commonly diagnosed in trauma patients due to the blood loss experienced during the injury. In acute blood loss after trauma, hemoglobin (Hb) and hematocrit (Hct) levels can appear normal due to the concomitant loss of both red blood cells (RBCs) and plasma^1^. Initially, the dominant feature of the critically injured is hypovolemia. The released vasopressin and other peptides cause body fluids to shift from the extravascular to the intravascular compartment, producing hemodilution^2^. Thus, hypovolemia gradually converts to anemia. A diagnosis of anemia is based on Hb levels of < 13 g/dL in men and 12 g/dL in women^3^, and the mean corpuscular volume (MCV) value, defined as the average size of RBCs, can be used to classify anemia as either microcytic, normocytic, or macrocytic^4^. Anemia due to acute blood loss, hemolysis, or malignancy is known as normocytic anemia (MCV, 80–100 fL)^1^.

However, MCV values are inevitably less useful in patients without anemia. The prevalence of macrocytosis in the general population is about 3%^5,6^, and the etiologic factors for macrocytosis range from vitamin B_12_ and folate deficiency and the use of chemotherapeutic and anticonvulsant agents to alcoholism, chronic obstructive pulmonary disease, and bone marrow disorder^5,7,8^. In some cases, the discovery of macrocytosis may not necessitate further testing or treatment because macrocytosis itself is not known to cause any direct complication^4^. Although the number of studies is limited, recent studies have shown that a high MCV is associated with poor outcomes in several diseases such as colorectal or esophageal cancer^9,10^, chronic kidney disease^11^, and ischemic stroke or coronary interventions^12,13^. However, information regarding the mechanisms underlying the relationship between MCV and these diseases is insufficient^5^.

Several studies have shown that the MCV value can be a useful indicator for the diseases mentioned above; however, to the best of our knowledge, it has not been studied in patients with trauma. Therefore, we conducted this study to investigate the association between MCV and 30-day mortality in critically ill patients with major trauma at a Level I trauma center.

## Methods

### Ethics approval

This study was approved by the Institutional Review Board (IRB no. 2022-05-021) of Chungbuk National University Hospital (CBNUH). The requirement for informed consent was waived by the Institutional Review Board (IRB no. 2022-05-021) of CBNUH due to the retrospective nature of the study. All methods were carried out in accordance with relevant institutional guidelines and regulations.

### Study design and patient selection

This single-site retrospective observational cohort study was conducted at a Level I trauma center at Chungbuk National University Hospital, South Korea. A total of 2020 trauma patients were admitted to the intensive care unit (ICU) between January 2016 and June 2020. Patients with the following characteristics were excluded: age $<$ 18 years, injury severity score (ISS) < 16, and transfer from other hospitals. Consequently, 802 patients were excluded and 1218 were enrolled. Patients were divided into two groups according to their anemia type, those with macrocytosis (n = 199) and those without macrocytosis (non-macrocytosis; n = 1019), and these groups were compared with respect to clinical outcomes with 30-day mortality.

This study was approved by the Institutional Review Board (IRB no. 2022-05-021), which waived the requirement for informed consent due to the retrospective nature of the study.

### Variables studied and definitions

The patient baseline characteristics included age; sex; initial systolic blood pressure (SBP); heart rate (HR); and trauma-related variables, such as the ISS, revised trauma score (RTS), and clinical outcomes such as duration of ICU stay, hospital stay, and the number of days on ventilatory support. Complete blood count (CBC) data was collected from blood samples obtained immediately in the emergency room.

Hypotension was defined as a systolic blood pressure < 90 mmHg. Anemia was diagnosed based on Hb levels < 13 g/dL in men and < 12 g/dL in women^3^. Elderly individuals were defined as those aged ≥ 65 years. An emergency operation was defined as surgeries performed within 24 h after admission to the emergency room. Transfusion within 4 h and 24 h with any blood component were expressed as transfusion_4 and transfusion_24, respectively. Macrocytosis was defined as an MCV value of ≥ 100 fL.

### Statistical analyses

Statistical analyses were performed using the R software version 4.1.0 (The R Foundation, Vienna, Austria) using additional packages: “moonBook”, “survival”, “ggplot2”, and “coxphf”. Listwise deletion was used for handling missing data. Categorical data are presented as frequencies with proportions and were compared using the chi-square or Fisher’s exact test. Continuous variables with normal distribution are expressed as mean and standard deviation, whereas those with skewed distribution are expressed as medians and interquartile range (IQR). The continuous variables were compared between groups using the Student’s *t*-test or Mann–Whitney *U* test. The initial macrocytosis and time to survival event were calculated using the Kaplan–Meier and log-rank tests, respectively. Statistical significance was set at p < 0.05. The Cox proportional hazards model was used for survival analysis, with adjustment for other potential confounding factors.

## Results

The patient baseline characteristics are presented in Table 1. Of the 1218 patients included in the study, initial macrocytosis was observed in 16.3% of patients (199 patients). The mean age was 57.6 years, and the age of patients in the macrocytosis group was significantly higher than that of patients in the non-macrocytosis group (61.2 vs. 56.9, *p* = 0.001). However, the proportion of elderly patients (aged $\ge$ 65 years) was not significantly different between the two groups (42.2% vs. 36.0%, *p* = 0.115). The initial SBP showed a significant difference between groups, and the proportion of hypotension was significantly higher in the macrocytosis group than in the non-macrocytosis group (34.7% vs. 26.6%, *p* = 0.006). However, ISS showed no significant difference (25.0 vs. 24.8, *p* = 0.704) between groups, and the AIS of the head and neck, chest, and abdomen also showed no significant difference.

**Table 1:** Baseline characteristics of trauma patients.

	Non-macrocytosis (n = 1019)	Macrocytosis (n = 199)	Total (n = 1218)	*p*-value
Age (years), mean ± SD	56.9 ± 18.5	61.2 ± 15.2	57.6 ± 18.1	0.001
Elderly, n (%)	367 (36.0%)	84 (42.2%)	451 (37.0%)	0.115
Sex, n (%)				0.044
Female	272 (26.7%)	39 (19.6%)	311 (25.5%)	
Male	747 (73.3%)	160 (80.4%)	907 (74.5%)	
Blunt trauma, n (%)	993 (97.4%)	196 (98.5%)	1189 (97.6%)	0.472
SBP, n (%)	117.1 ± 43.0	106.3 ± 51.6	115.4 ± 44.7	0.006
Hypotension, n (%)	255 (25.0%)	69 (34.7%)	324 (26.6%)	0.006
ISS, mean ± SD	24.8 ± 8.4	25.0 ± 8.2	24.8 ± 8.4	0.704
AIS_H&N, median (IQR)	3.0 (0.0–4.0)	3.0 (0.0–5.0)	3.0 (0.0–4.0)	0.884
AIS_Chest, median (IQR)	2.0 (0.0–3.0)	2.0 (0.0–3.0)	2.0 (0.0–3.0)	0.813
AIS_Abdomen, median (IQR)	0.0 (0.0–2.5)	0.0 (0.0–2.5)	0.0 (0.0–3.0)	0.899
RTS, median (IQR)	7.5 (6.0–7.8)	6.9 (5.0–7.8)	7.5 (6.0–7.8)	0.002

*Elderly* age $\ge$ 65 years, *SBP* systolic blood pressure, *Hypotension* SBP < 90 mmHg, *ISS* injury severity score, *AIS* abbreviated injury scale, *H&N* head and neck, *RTS* revised trauma score, *IQR* interquartile range.

**Figure 1:**
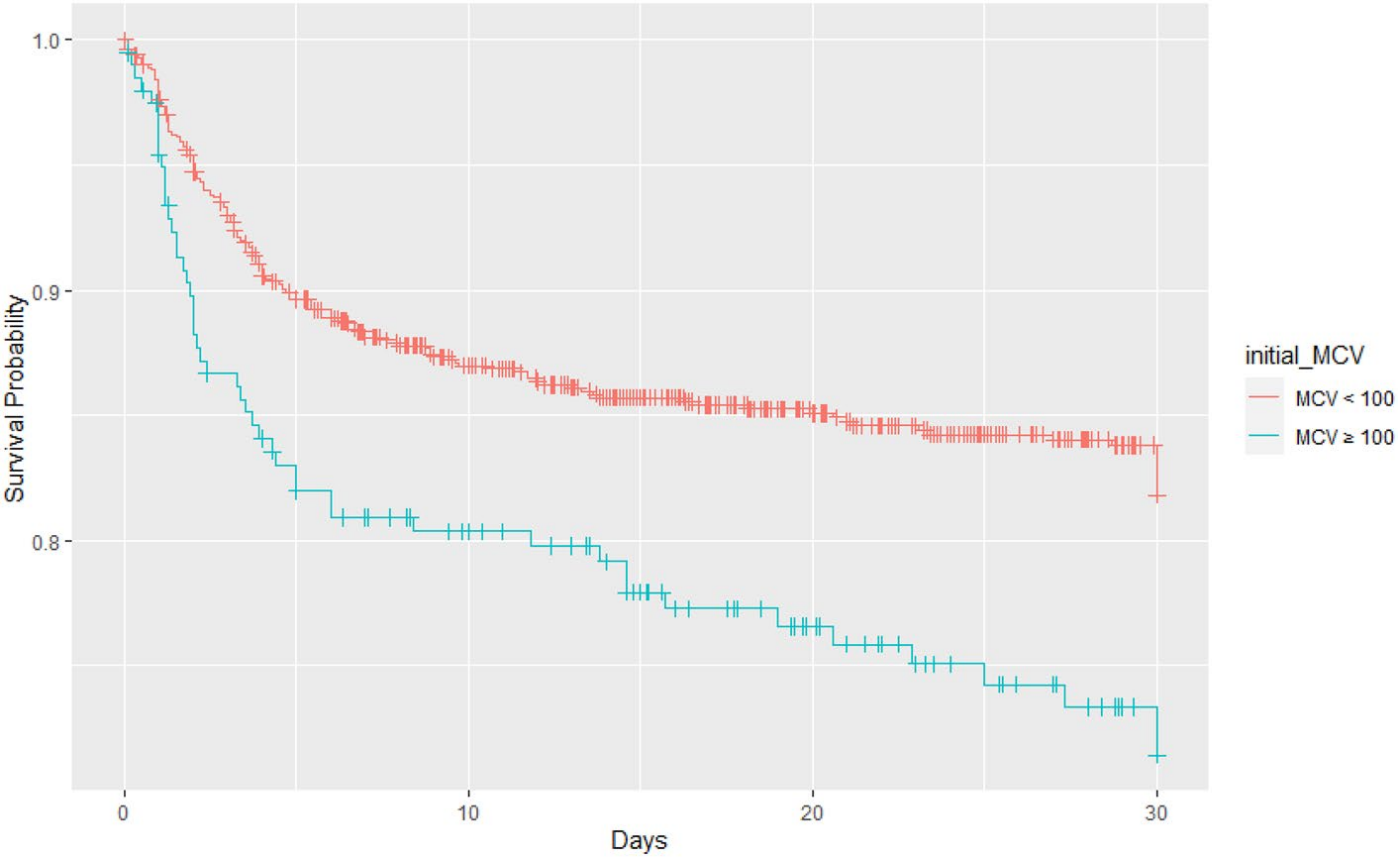
Kaplan–Meier survival curve. Survival probability for 30-day mortality in the macrocytosis and non-macrocytosis groups (*p* < 0.001, log-rank test).

The macrocytosis group underwent more transfusions than did the non-macrocytosis group during the initial 4 h (46.7% vs. 35.0%, *p* = 0.002) and 24 h (57.8% vs. 48.1%, *p* = 0.015) after admission. The proportion of emergency operations and the duration of ICU stay showed no significant differences between the two groups. The 30-day mortality rate was 24.1% in the macrocytosis group, which was higher than that in the non-macrocytosis group (14.5%) (Table 2). The 30-day survival curve for the macrocytosis group compared with that for the non-macrocytosis group is shown in Fig. 1. Both groups had acutely increased mortality for the initial 10 days, after which the mortality curves increased more slowly.

**Table 2:** Comparison of clinical parameters between the initial macrocytosis and normocytosis groups.

	Non-macrocytosis (n = 1019)	Macrocytosis (n = 199)	Total (n = 1218)	*p*-value
Emergency operation, n (%)	369 (64.0%)	79 (70.5%)	448 (65.0%)	0.219
Hb (g/dL), mean ± SD	12.7 ± 2.3	12.0 ± 2.4	12.6 ± 2.3	< 0.001
Anemia, n (%)	420 (41.2%)	111(55.8%)	531(43.6%)	< 0.001
RBC (10^6^/uL), mean ± SD	4.1 ± 0.7	3.5 ± 0.7	4.0 ± 0.7	< 0.001
MCV (fL), mean ± SD	93.2 ± 4.4	103.9 ± 4.1	95.0 ± 5.9	< 0.001
Transfusion_4, n (%)	357 (35.0%)	93 (46.7%)	450 (36.9%)	0.002
Transfusion_24, n (%)	490 (48.1%)	115 (57.8%)	605 (49.7%)	0.015
pRBC_4, mean ± SD	1.6 ± 3.5	2.4 ± 4.3	1.7 ± 3.6	0.011
FFP_4, mean ± SD	0.7 ± 2.0	1.2 ± 2.9	0.8 ± 2.2	0.016
Ventilator care, n (%)	533 (52.3%)	121 (60.8%)	654 (53.7%)	0.034
LoICU (days), n (%)	8.5 ± 14.0	9.7 ± 13.5	8.7 ± 13.9	0.271
30-day mortality, n (%)	148 (14.5%)	48 (24.1%)	196 (16.1%)	0.001

*Hb* hemoglobin, *RBC* red blood cell, *MCV* mean corpuscular volume, *Transfusion_4* transfusion within 4 h, *Transfusion_24* transfusion within 24 h, *pRBC_4* count of packed red blood cell within 4 h, *FFP_4* count of fresh frozen plasma within 4 h, *LoICU* length of intensive care unit stay.

**Figure 2:**
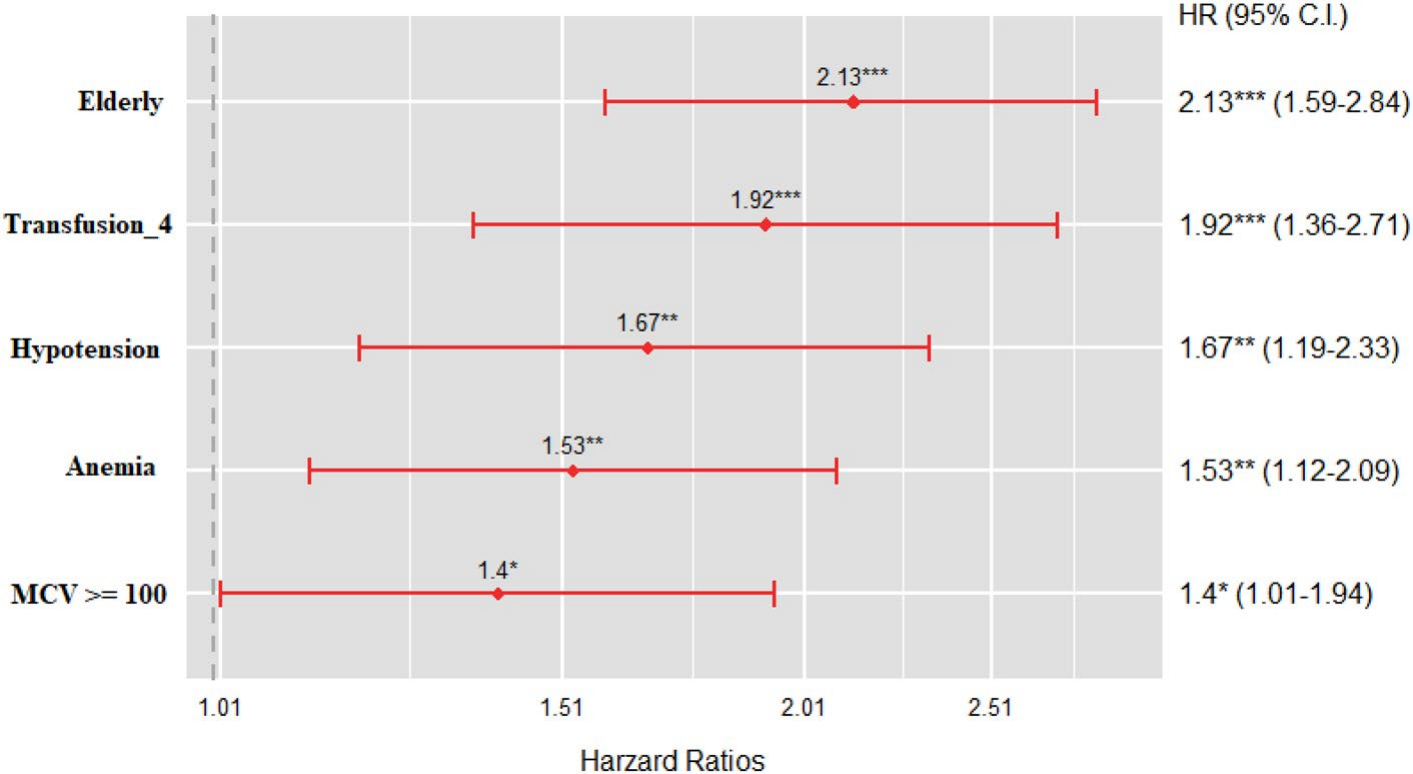
Cox regression proportional hazards analysis. Hazard ratios for significant variables for 30-day mortality (*HR* hazard ratio, *CI* class interval).

To identify factors associated with 30-day mortality, Cox regression proportional hazards analysis was performed using several binary compound factors such as age, transfusion_4, hypotension, anemia, and macrocytosis (Fig. 2). All of these factors were significantly associated with 30-day mortality in severely injured trauma patients.

## Discussion

The primary finding of this study was that initial macrocytosis was associated with 30-day mortality in elderly patients with major trauma, along with factors such as transfusions within 4 h of admission, hypotension, and anemia.

In our cohort, the prevalence of macrocytosis was 16.3%, and it was associated with increased 30-day mortality compared to that in the non-macrocytosis group. This finding may be explained by hypoperfusion due to major trauma in patients with macrocytosis. The relationship between MCV and Hb was established almost a century ago^14^, which was validated in a more recent study where MCV was shown to vary in a strict linear relationship with the average Hb content of RBCs^15^. Accordingly, larger erythrocytes (macrocytes) accommodate a greater amount of Hb compared to smaller erythrocytes^16^. In our study, no statistical differences were found in the mean MCV values between the anemic and non-anemic groups (Supplementary Table S1); however, between the hypotension and non-hypotension groups, the mean MCV value and proportion of macrocytosis showed significant differences (Supplementary Table S2).

Multiple major traumas can cause systemic inflammatory response syndrome (SIRS) via hormonal, metabolic, and immunological mediators^17,18^. This response occurs immediately after major trauma and aggravates the initial damage caused by hypoperfusion and reperfusion^19^. This metabolic response is associated with increased oxygen demand in the tissues^17^. Consequently, to compensate for the increased oxygen consumption, the body reacts with tachycardia, increased cardiac output, increased respiratory rate, and vasodilatation^17,20^. As mentioned previously, anemia due to acute blood loss, hemolysis, or malignancy is known as normocytic anemia^1^. In most cases, the etiology of macrocytosis may involve abnormal RBC development, abnormal RBC membrane composition, increased reticulocyte count, or a combination of these factors^21^. Although the results of the high proportion of macrocytosis in our study are not explained by any particular etiologies, it seems reasonable that erythrocytes are enlarged to respond to the increased oxygen demand; however, biological studies are required to confirm this.

Erythrocytes transport oxygen and carbon dioxide, and their membranes have systemic antioxidant properties since they are potentially exposed to oxidative stress^22^. Macrocytosis is a structural and functional abnormality of the erythrocyte membrane^12,21^. Oxidative stress resulting from the overproduction of reactive oxygen species is considered to contribute to the development and progression of cardiovascular disease^23^. Therefore, MCV is not only an indicator of anemia but also a marker of inflammation and endothelial function^9^. Studies investigating the underlying pathophysiology of macrocytosis in patients with trauma are required in the future.

Our study had several limitations. First, it was a retrospective, observational, single-center study. Second, we could not account for confounding factors such as chronic hepatitis, chronic renal disease, or alcohol abuse on MCV changes, and the influence of these factors should be addressed in future studies. Third, we could not always distinguish between patients admitted through the emergency room and those transferred from other hospitals, which could have altered the MCV values. Finally, macrocytosis is indicated by a high MCV on the CBC test and is confirmed by peripheral blood smear analysis. Thus, further study by peripheral blood smear is required.

## Conclusion

This study revealed that the prevalence of initial macrocytosis was 16.3% in major trauma patients and that it was independently associated with 30-day mortality along with other contributing factors such as age ($\ge$ 65 years), transfusions within 4 h, hypotension, and anemia. Further studies involving peripheral blood smear and biological mechanisms are warranted to confirm that the increased MCV value reflects the enlargement of erythrocytes.

### Supplementary Information

Supplementary Table 1.

Supplementary Table 2.

## Data Availability

The datasets generated during and/or analyzed during the current study are not publicly available due to our institutional policy but are available from the corresponding author on reasonable request.
