# Supplementary material for: Mean corpuscular volume as a prognostic factor for 30-day mortality in major trauma patients: a retrospective cohort study
Source: Sci Rep. 2024 Feb 17;14:3951. doi: 10.1038/s41598-024-54057-1 (PMC10873376; doi:10.1038/s41598-024-54057-1)
Supplement: Supplementary file 1 — Supplementary Table 1. [file 41598_2024_54057_MOESM1_ESM.docx]

**Supplementary Table S1.** Initial MCV statistics for trauma patients with anemia and those without anemia

|  | No anemia  (n=687) | Anemia  (n=531) | Total  (n=1218) | *p*-value |
| --- | --- | --- | --- | --- |
| **Hb (g/dL),** mean ± SD | 14.2 ± 1.1 | 10.6 ± 1.8 | 12.6 ± 2.3 | <0.001 |
| **MCV (fL),** mean ± SD | 94.8 ± 4.7 | 95.2 ± 7.2 | 95.0 ± 5.9 | 0.238 |
| **Macrocytosis, n (%)** | 88 (12.8%) | 111 (20.9%) | 199 (16.3%) | <0.001 |

**Hb**, hemoglobin; **MCV**, mean corpuscular volume
