# Supplementary material for: Mean corpuscular volume as a prognostic factor for 30-day mortality in major trauma patients: a retrospective cohort study
Source: Sci Rep. 2024 Feb 17;14:3951. doi: 10.1038/s41598-024-54057-1 (PMC10873376; doi:10.1038/s41598-024-54057-1)
Supplement: Supplementary file 2 — Supplementary Table 2. [file 41598_2024_54057_MOESM2_ESM.docx]

**Supplementary Table S2.** Initial MCV statistics for trauma patients with hypertnesion and those without hypotension

|  | No hypotension (SBP $\geq$ 90 mmHg)  (n=894) | Hypotension  (SBP < 90 mmHg)  (n=324) | Total  (n=1218) | *p*-value |
| --- | --- | --- | --- | --- |
| **Hb (g/dL),** mean ± SD | 12.9 ± 2.1 | 11.7 ± 2.4 | 12.6 ± 2.3 | <0.001 |
| **MCV (fL),** mean ± SD | 94.5 ± 5.8 | 96.2 ± 6.0 | 95.0 ± 5.9 | <0.001 |
| **Macrocytosis, n (%)** | 130 (14.5%) | 69 (21.3%) | 199 (16.3%) | 0.006 |

**SBP**, systolic blood pressure; **Hb**, hemoglobin; **MCV**, mean corpuscular volume
